# Commercial Seaweed Liquid Extract as Strawberry Biostimulants and Bioethanol Production

**DOI:** 10.3390/life13010085

**Published:** 2022-12-28

**Authors:** Mohamed Ashour, Ahmed Said Al-Souti, Shimaa M. Hassan, Gamal A. G. Ammar, Ashraf M. A.-S. Goda, Rania El-Shenody, Abd El-Fatah Abomohra, Ehab El-Haroun, Mostafa E. Elshobary

**Affiliations:** 1 National Institute of Oceanography and Fisheries, NIOF, Cairo 11516, Egypt; 2 AL Hail Aquaculture Unit, Department of Marine Science, Fisheries College of Agriculture and Marine Science, Sultan Qaboos University, Muscat 123, Oman; 3 Department of Vegetable Crops, Faculty of Agriculture (El-Shatby), Alexandria University, Alexandria 21545, Egypt; 4 Biotechnology Unit, Plant Production Department, Arid Lands Cultivation Research Institute, The City of Scientific Research and Technological Applications, Alexandria 21934, Egypt; 5 Botany Department, Faculty of Science, Tanta University, Tanta 31527, Egypt; 6 New Enery and Environmental Laboratory (NEEL), School of Architecture and Civil Engineering, Chengdu University, Chengdu 610106, China; 7 Fish Nutrition Research Laboratory, Animal Production Department, Faculty of Agriculture, Cairo University, Cairo 11562, Egypt

**Keywords:** True Algae Max (TAM^®^), seaweed extract, *Ulva lactuca*, *Jania rubens*, *Pterocladia capillacea*, biorefinery, bioethanol, biofuels, strawberry

## Abstract

Seaweeds are increasingly intriguing as a sustainable source of bioactive compounds. They have applications in agriculture, fuels, feed, and food products. To become a cost-competitive product with zero waste, a biorefinery approach is applied, where several products are valorized at the same time. True-Algae-Max (TAM^®^) has been investigated for its ability to improve the yield and nutritional facts of a strawberry plant. Three concentrations of TAM (0, 50, and 100%) were examined by foliar spray in 2017 with 50% NPK chemical fertilizer. Results indicated that growth, yield, chlorophyll, and potassium content were significantly improved by TAM treatments. TAM50 % resulted in maximum root length, leaf area, plant fresh weight, fruit weight, and yield with an increase ranging from 10 to 110% compared to control. Compared to the NPK control, strawberries grown with TAM50% improved total soluble solids (TSS) from 7.58 to 10.12% and anthocyanin from 23.08 to 29.42 mg CGE 100 g^−1^. Noteworthily, this reduced total sugar, and total phenolics were boosted by TAM applications, while non-reducing sugar was reduced compared to control. On the other hand, whole seaweed biomass and TAM residuals were used for bioethanol production by acid scarification. The maximum bioethanol yield was observed in residual biomass (0.34 g g^−1^ dw), while the whole seaweed biomass showed only 0.20 g g^−1^ dw. These results proved the biorefinery concept of using seaweed extract as a biostimulator and bioethanol production.

## 1. Introduction

Food and energy are two faces of the same coin. With the world’s ever-increasing population and increasing demand for food and energy, there is growing pressure to produce sufficient food and energy [1,2]. However, the depletion of essential nutrients required for crop growth as well as fossil fuel resulted in a real problem facing human beings. To address the pressures correlated with increasing agronomic productivity, farmers have extensively used chemical fertilizers and pesticides to boost agriculture productivity. However, these dangerous chemicals threaten all kinds of life and the whole biosphere [3,4]. Hence, there is a great demand for developing an effective method to lessen reliance on artificial fertilizers [1]. The term “biostimulants” refers to biologically derived substances or microorganisms that stimulate natural plant processes when applied to plants through root soak, foliar spray, or a combination of both. Biostimulants are responsible for efficient nutrient use, growth, and increasing tolerance to abiotic and biotic stress [5]. Biostimulants are composed of mixtures of bioactive compounds that could offer a potential alternative to harmful agrochemical fertilizers. They have the ability to lower the usage rates of artificial fertilizers by inducing plant performance, growth, and total yield, as well as helping plants to survive under biotic and abiotic stresses without any negatives [4,6,7,8]

Seaweeds are marine organisms rich in numerous phytochemical bioactive compounds that can be used for commercial applications [9,10,11,12,13,14]. Seaweeds have applications in fuels [15,16,17], feed [18,19,20], bioremediation [21,22,23,24], and food products [25,26]. The Egyptian coasts of the Mediterranean [27,28,29] and Red Sea [30,31] offer a diverse range of wild seaweed genera that may renew and be harvested all year. Despite the Mediterranean having just 0.82% of the world’s ocean’s surface area and 0.32% of its volume, it is the biggest and deepest confined sea and a marine biodiversity hotspot [32,33]. The Mediterranean Sea is home to around 1124 seaweed species, at least 20% of which are indigenous [34,35] and which are represented throughout the year. However, this area needs to be taken in our consideration, as it faces anthropogenic activity, ocean acidification [36,37], microplastic pollution [38], etc. According to the previous stress and pollution, these seaweeds accumulate several primary and secondary metabolites that can be used in several applications, such as carbohydrates, making them a good bioethanol source. Red alga, *Pterocladia capillacea*, *Jania rubens*, and green alga, *Ulva lactuca,* are the most prevalent native seaweed species along Alexandria’s Mediterranean shore, Egypt [8,27,29]. The biochemistry, mineral content, antioxidant activity, and antimicrobial activity of these organisms are well studied [27,29,36]. Seaweeds have been used as biostimulants for centuries, but have become more widely accepted over the past 15–20 years, particularly in organic agriculture [1,4,7,8,35,39,40]. Seaweed extracts provide a widely available source of nutrients and biomolecules that enhance both soil and plant. Additionally, seaweed biostimulants have been shown to improve stress tolerance, mineral uptake, growth, performance, yield, reduce seed dormancy, and enhance plant roots and fruit quality [41]. The features of commercial extracts, of course, depend on the algae species and the procedures used to prepare them [42]. Although there are different ways of seaweed extract, foliar spray application proved effective to improve the yield and performance of many commercial plants [1,8].

Despite the raw materials for lignocellulosic bioethanol being inexpensive, renewable, and plentiful, the manufacture of second-generation bioethanol is expensive due to the high resistance of lignocellulosic raw materials [43]. As a result, with the energy crisis and environmental pollution substantially impeding contemporary society’s sustainable growth, seaweed biomass as a raw material for bioethanol synthesis is unquestionably a sustainable and eco-friendly source for renewable biofuel production [44,45]. In spite of the fact that more than 100 algae-to-fuel firms have been established throughout the world, particularly in the past few years, no commercial plant has yet to be constructed. As with any other industrial process, the commercialization of algal bioethanol is based on the method’s economics. Various obstacles in the technology of algal bioethanol production must be addressed before it can be produced on a wide scale and commercially [45]. The most expensive phase in the manufacture of algal bioethanol, pretreatment, is expected to account for around 33% of the entire cost. Thus, a pretreatment process must be simple and prevent large chemical consumption and energy demands [46].

Food and energy can be strategically integrated to increase gross productivity while reducing cost production, losses, and negative environmental impact. However, seaweed-based industrial wastes have yet to be discovered. The proper use of these marine organic wastes for useful products will be effective, smart, and cost-effective techniques. Seaweed residues, after extracting pigments, lipids, proteins, and phycocolloids, still contain energetic compounds, such as cellulose, and some left-out polymers were found as potential feedstock for sugar hydrolysis to ethanol production [47,48]. On the other hand, using natural biomass wastes as an alternative fuel is favorable because the quantity of CO_2_ used during biomass production is released after combustion, resulting in no net CO_2_ buildup in the atmosphere [48]. Ethanol production has been reported from all groups of seaweeds, either the whole thallus [49] or apart from whole seaweeds, such as industrial waste, floating residues, and spent biomass [48]. The potential for ethanol generation from seaweeds is based on the high carbohydrate content reach to 60% dry weight and ethanol conversion efficiency reached to 90% [39]. The present study is aimed to minimize the agricultural chemical footprint by using seaweed biostimulants considering the vegetative growth, yield, nutrient contents, and bioactive compounds and production of ethanol by utilizing residual biomass using baker’s yeast through a biorefinery concept.

## 2. Materials and Methods

### 2.1. Seaweed Liquid Extract Methods

TAM^®^ is a commercial seaweed liquid extract submitted as a patent [50]. The preparation of TAM^®^ was as described by Ashour et al. [12]. In detail, three seaweed species, *Ulva lactuca* (Chlorophyceae), *Jania rubens,* and *Pterocladia capillacea* (Rhodophyceae), were used in preparing and producing TAM^®^. The selected species were collected in the 2016 summer season from the rocky site (31°16′16.0″ N, 30°10′28.0″ E) of Abu-Qir Bay, the Mediterranean Coast of Alexandria, Egypt. After being harvested, the epiphytic and waste materials were removed, then samples were washed, air-dried, powdered, and, finally, kept in plastic bags at room temperature for further analysis. Phytochemical, physical, chemical, and biochemical analyses of crude TAM^®^ were conducted and estimated, as described by Ashour et al. [12]

### 2.2. Bioactive Constituents and Antioxidant Activities of TAM^®^

The bioactive contents of crude TAM^®^,^,^ including ascorbic acid, flavonoid content, and total phenolic compounds, have been estimated, as described by El-Shenody et al. [31]. In addition, total antioxidant activity (mg g^−1^) and DPPH inhibition (%) were measured in TAM extract according to El-Shenody et al. [31].

### 2.3. Bioethanol Production from Residual Biomass Compared to the Whole Biomass

For biorefinery purposes, the whole seaweed and residual biomass after obtaining TAM extract was further investigated for bioethanol production by acid hydrolysis and fermentation process. About 5 g of each biomass was hydrolyzed in 5% of a 98% H_2_SO_4_ (1:25, *w/v*). The mixture was heated at 121 ℃ for 30 min and 1.5 Bar; then, the total reducing sugars were obtained by sieving the mixture through cheesecloth [16]. Soluble reducing sugars were estimated by the micro-phenol-sulfuric acid method [51]. The hydrolysates was subjected to batch fermentation in a 250 mL dark bottle containing 100 mL of the hydrolysates, 0.9 g L^−1^ ammonium sulfate, and 0.375 g L^−1^ yeast extract at pH 4.6 [52]. The mixture was inoculated with 5 g of dry active granules of Angel yeast (*Saccharomyces cerevisiae* CECA) purchased from Angel Co., Ltd. The culture was incubated at 30 ℃ for 96 h on a shaking incubator at 120 rpm. Residual sugars and bioethanol production were estimated by aliquoting 2 mL of culture at 6 h intervals during fermentation. As mentioned before, the total residual sugars were measured following the nitrophenol–sulfuric acid method, while bioethanol was quantitatively estimated by the potassium dichromate method [53]. The ethanol concentration was monitored based on the ethanol standard curve using 0.5–5.0 g L^−1^ of ethanol.

### 2.4. Field Experiment

#### Experimental Design

The field experiment was carried out during the autumn season of 2017 at Omar Makram Village (30°34′27.1″ N 30°44′23.6″ E), Badr Center, Beheira Governorate, Egypt. Before the trial started, soil samples were taken at depths ranging from 15 to 30 cm, and then were assessed according to Sparks et al. [54] at the Central Lab., Fac. of Agriculture, Alexandria Univ., Egypt. Table 1 showed the chemical and physical specifications of soil samples.

The study was conducted under a drip irrigation system to investigate the effects of covenantal mineral chemical fertilizers and seaweed liquid extract (TAM^®^) on vegetative growth characters, flowering traits, fruit yield, and quality of strawberries (Fortuna™, FL 01 116 cv.) that were obtained from the Non-Traditional Crops Research Station Nubaria, El-Beheira Governorate, Egypt and transplanted into the field on 25 October of the growing season. The experiment continued until the 15 May 2018. The experimental layout included three replications for each treatment, and it utilized a RCPD system in a randomized complete blocks design.

The strawberry plant transplants were cultivated in raised beds of 15–22 cm in height and 120 cm wide. Three treatments were conducted in this study, and each treatment was applied in 2100 m^2^ (three replicates of 700 m^2^ for each). The transplants were planted under the drip irrigation network that was designed for this study. The drip irrigation network consisted of a lateral GRs of 16 mm in diameter, with emitters at a 0.5 m distance, allocating two laterals for each ridge. The emitters had a discharge rate of 4 l h^−1^. During soil preparation, at the rates of 41 kg N, 46.5 kg P_2_ O_5,_ and 72 kg K_2_O as ammonium sulphate (20.5% N), calcium super phosphate (15.5% P_2_O_5_), and potassium sulphate (48% K_2_O) were added. The rest of the nitrogen, phosphorus, and potassium fertilizers amounts were added through the drip irrigation system three times per week, at the rates of 200 kg N Fed^−1^ from ammonium nitrate (33.5% N), 80 L P_2_ O_5_ Fed^−1^ from phosphoric acid (80% P_2_O_5_), 120 kg K_2_O as potassium sulfate (48% K_2_O), and a microelement (Fe, Mn, and Zn), which were all added as foliars sprayed three times monthly after transplanting. This method is commonly used in the commercial production of a strawberry plant according to what is outlined by the Ministry of Agriculture and Reclamation of Egypt. In the current experiment, three levels of crude TAM^®^ (TAM0, TAM50, and TAM100) were applied: TAM0: 0% (0 mL L^−1^) of crude TAM^®^, as a control 100% NPK classical chemical fertilizer; TAM50 (500 mL Fed^−1^) of crude TAM^®^ as a 50% replacement of the recommended NPK classical chemical fertilizer that added through the drip irrigation system during the growing season; and TAM100: (100 mL Fed^−1^) of crude TAM^®^ as a 100% replacement of the recommended NPK traditional chemical fertilizer. The recommended NPK classical chemical fertilizer and/or the three additional levels of crude TAM^®^ were added through the drip irrigation system three times per week.

### 2.5. Tested Parameters

After 130 days from transplanting, a random sample of ten strawberry plants was taken from each replicate to measure plant length, plant fresh weight, leaf area, root length, and total chlorophyll content. Random samples of the youngest expanded mature leaves of plants were randomly collected from each replicate to determine the total nitrogen, potassium, and phosphorus contents of leaves. The youngest mature leaves were collected, washed with distilled water, weighed, and then oven-dried at 70 °C until constant weight. The dried leaf materials were ground and homogenized, as well as wet digested using concentrated sulfuric acid and H_2_O_2_. Total nitrogen, potassium, and phosphorus in leaves of strawberry plants were determined calorimetrically using a spectrophotometer at 662 and 650 nanometers according to Evenhuis [55] and Murphy and Riley [56]. The marketable size of strawberry fruit was counted and weighed one to three times a week from March to May 2018. Harvesting a marketable size of strawberry fruits was performed 130 days after transplanting, on the 25th of October. From each replicate, the fruits were harvested randomly from ten plants, collected, and, then, ten fruits were randomly taken so as to measure fruit weight (g), and fruit yield plant^−1^ (g) was then calculated. The total soluble solids (TSS %) of collected fruits were determined according to the methods described by AOAC [57]. Titratable acidity (TA) was determined by titration using a standard 0.1 M NaOH solution to a pH of 8.2, according to Zenebon [58]. Anthocyanin content (mg CGE 100 g^−1^) was determined according to the method of Giusti and Wrosltad [59]. Total phenolic content (mg GAE 100 g^−1^) was determined by spectrophotometry by using the Folin–Ciocalteau phenol reagent according to the method described by Kumar and Balamuruga [60]. Total sugar percent (TS) and reducing sugar (RS) percent were determined, as described by AOAC [57], while non-reducing sugar (NRS) percent was calculated as the difference between the TS and RS.

### 2.6. Statistical Analysis

Statistical analysis was performed using analysis of variance (ANOVA). Differences among means were considered significant at *p* < 0.05, and multiple ranges of post hoc comparisons were performed using the least significant difference (LSD) to resolve the differences among the means of replication according to Duncan using the SPSS program.

## 3. Results and Discussion

Current disruptions in agricultural economies and the agro-environment have raised questions about future food and energy security and highlighted the urgent need for sustainable practices that do not threaten environmental assets. The biorefinery approach is one of the key focuses of current research activities that provide a smart utilization of the whole biomass with zero residues. In particular, one of the main challenges is finding new methodologies that can stimulate the whole plant’s performance and quality, along with producing sustainable energy sources using residual biomass. In this context, seaweed extracts are becoming more widely accepted in agriculture as plant biostimulants due to several beneficial effects against nutrient depletion of the soil [8]. The purpose of this study was to compare the effect of foliar application of seaweed commercial extract (TAM^®^) on strawberry growth, yield, and fruit quality to that of chemical NPK fertilizer and using the residual biomass after obtaining TAM^®^ extract for bioethanol production. Physical and chemical analyses of TAM^®^ extract have been previously analyzed and confirm the presence of a mixture of nutrients, as well as macro- and micronutrients, in addition to different bioactive compounds while considering the neglected amount of heavy metals that work in simultaneous pattern to improve the plant growth, performance, and yield [4,7]. For this purpose, different combinations of serial concentrations from NPK and TAM^®^ mixed in ratios of 0% (TAM0), 50% (TAM50), and 100% pure TAM^®^ (TAM100) were afterward prepared to survey the effectiveness of the foliar spray application versus control using 0% TAM and 100% conventional NPK mineral fertilizer.

### 3.1. Bioactive Compounds and Antioxidant Activities of Crude TAM^®^

Crude extract of TAM^®^ has been tested for its bioactive compounds and antioxidant activities. Regarding bioactive compounds, phenolic compounds were the leading bioactive compounds at 101.67 mg g^−1^, flavonoid compounds were in the second order of 2.60 mg g^−1^, and ascorbic acid represented only 1.66 mg g^−1^. These bioactive constituents showed a great total antioxidant capacity, reaching 54.52 mg g^−1^ and a DPPH inhibition of 70.33%.

### 3.2. Growth and Vegetative Performances

Table 2 shows the effect of different treatments of crude TAM^®^ on berry growth characterization. The treatment with TAM^®^ positively influenced the vegetative growth and performances of strawberry plants. More in detail, TAM50 and TAM100 substantially increased plant fresh weight, leaf area, and root length, while there is no significant difference in plant length among the three treatments at *p* < 0.05. Compared to the control, TAM50 showed the highest plant fresh weight, leaf area, and root length with an increase of 10%, 25%, and 110%, respectively, compared to the control of TAM0.

Results confirmed that TAM100 significantly increased the concentration of leaf chlorophyll content (34.37 mg 100 g^−1^ fresh weight (FW)) and total leaf-K (1.34%), followed by TAM50, while TAM_0_ showed the lowest content. On the other hand, neither the total leaf-N, nor leaf P, were influenced by the treatments. The improved vegetative growth means that more mineral nutrients and photoassimilates are available to the various plant organs in the treated plants. The treated plants had a more evolved root system in terms of mineral nutrients, which may positively affect nutrient uptake [61]. Furthermore, the TAM^®^ extract’s composition, which includes fatty acids, rhodopin, phytol, milbemycin-oxime, phytol, nonadecane, and silicon-boron compound, makes it an ideal product for maximizing vegetative growth and enhancing fruit quality and size [4,7]. In this context, Changlin et al. confirmed that strawberry conches fed with three different seaweeds showed a high survival rate and a high increase in dry weight.

The highest specific growth rate of strawberries was observed in *Undaria pinnatifida* treatment [62]. It was observed that applying commercial seaweeds extract (Seasol^®^) from *Duvillaea potatorum* and *Ascophyllum nodosum* can improve root growth by increasing the density of secondary roots by up to 22%, as well as increasing transplant (runner) by 8–19% in two strawberry cultivars (‘Albion’ and ‘Fortuna’) [63]. According to studies by Spinelli et al. [61] and El-Miniawy et al. [64], applying biostimulant products that contain extracts from the seaweed *Ascophyllum nodosum* increased strawberry root dry weight by 35–130%. Results confirmed that TAM100 significantly increase the concentration of leaf chlorophyll content (34.37 mg 100 g^−1^ FW) and total leaf-K (1.34%), followed by TAM50, while TAM0 showed the lowest content. On the other hand, neither the total leaf N, nor leaf P, were influenced by the treatments.

### 3.3. Yield and Reproductive Performances

**Figure 1:**
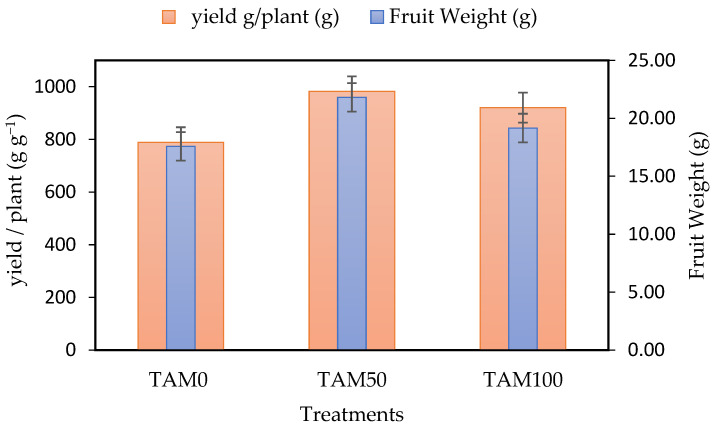
Fruit weight (g) and yield (g plant^−1^ g^−1^) under different TAM^®^ concentrations.

The effects of TAM^®^ on fruit productivity and strawberry quality parameters were also evaluated (Figure 1). TAM^®^ has substantially increased the average weight of fresh berries to 21.80 g by 24% for TAM_50_ and 9% for TAM100 compared to the control of 17.58 g. Fruit yield, expressed as grams of berry per plant, was significantly increased by the TAM50 to 982.33 g plant^−1^ g (up to 25%) and TAM100 (up to 17%) compared to the control of 789 g plant^−1^ g. Consequently, the increment in fruit yield was attributable to the higher fruit weight and the greater number of fruits produced by treated plants (Table 2). The obtained results of vegetative growth parameters agree with those reported by using the TAM on *Cucumis sativus* [4] and *Eruca vesicaria* (L.) Cav. [7]. According to Bogunovic et al. [65], seaweed biostimulants can help strawberry plants to grow and survive in the severe late spring freeze. They observed that all of the studied strawberry cultivars treated with a biostimulant had higher frost resistance, and the increased biostimulant concentration increased fruit weight [65]. Algreen—seaweed extract improved strawberry vegetative growth as well as fruit yield and quality of fruit full-grown in field conditions [64]. Moreover, soluble seaweed extract of *Ascophyllum* sp. increased bacterial community in greenhouse and field soil samples of treated strawberry cultivars [39], which may enhance the root length and mineral uptake.

Different parameters have been monitored to estimate the fruit quality, including total soluble solids (TSS %), titratable acidity (TA), TSS/TA, anthocyanin content, total sugar, reducing sugar, non-reducing sugar, and total phenolic content. The TSS content of strawberries refers to the amount of total soluble solids in the fruit pulp, such as carbohydrates, vitamins, amino acids, and pectin, while the TA reflects the acid concentration [66]. The current study confirmed that the TSS/TA balance is linked to fruit taste [67]. TAM50 was the leading treatment that improved TSS% and anthocyanin content from 7.58 to 10.12% and from 23.08 to 29.42 mg CGE 100 g^−1^, respectively, compared to the control. Since TAM^®^ did not increase TA % in the fresh berry, TSS/TA ratio has been significantly elevated by TAM50 (up to 62%) and TAM100 (up to 53%), which may be due to the higher TSS content (Table 3). A high value of this ratio is an obvious sign of an excellent combination of sugars and acidity, characterizing soft-tasting fruits [68]. These results are consistent with the effect of applying strawberry plants with amino acids (peptone), which significantly increased TSS, vitamin C, and total sugar content of fruits compared to the control treatment [69]. Another study on watermelon demonstrated that all treatments of Acadian^®^ seaweeds extract showed significant improvement in TSS and TSS/TA [68].

Moreover, total phenolic content has been increased by TAM treatment compared to control with an increase of 92% for TAM100 and 42% for TAM50. TAM50 extract has improved anthocyanin content from 23.08 to 29.42 mg CGE 100 g^−1^. Several studies indicated that a foliar application of seaweed extract to the leaves of strawberries increased the nutrient content of anthocyanins [70,71,72]. In another study, seaweed extract significantly improved the content of anthocyanins in all seeds of the two tested bean cultivars of *Phaseolus vulgaris* L [73]. A foliar spray of seaweed extract to the leaves of *Vitis vinifera*, after full bloom, improved the nutrient content of grapevines, specifically the accumulation of anthocyanins and phenolics [74,75]. In another study, commercial Kelpak^®^ seaweed extract significantly improved the content of anthocyanins in all seeds of the two tested bean cultivars of *Phaseolus vulgaris* L. Interestingly, strawberries treated by TAM^®^ contained significant amounts of total phenol compounds compared to those obtained from the control. In this context, *Solanum lycopersicum* treated with brown seaweed extract of *Ecklonia maxima* showed an increased yield and content of minerals, lycopene, total phenols, and ascorbic acid [76]. Our results on TAM^®^ were consistent with Hassan et al., who showed the same results of improving the total phenolic compounds in *Eruca vesicaria* Cav. [7] and *Cucumis sativus* [4] using TAM^®^ as a foliar spray.

**Figure 2:**
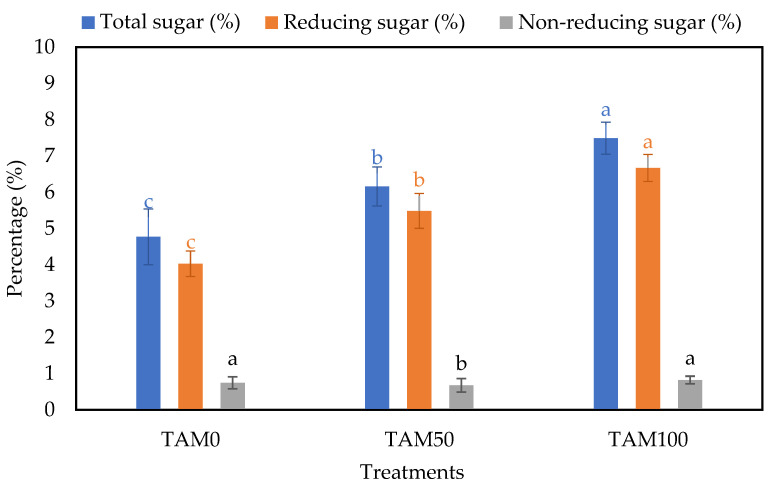
TAM total sugar, reducing, and non-reducing sugar content. Different letters (a > b > c) in each colomn indicate significant differences (*p* < 0.05).

As far as the biochemical content, the TAM^®^ application increments the total sugar, reducing and non-reducing sugar content (Figure 2). The highest increase was observed in TAM100 followed by TAM50 with an increase of 1.57 and 1.29 folds for total sugar content and 1.66 and 1.36 folds for reducing sugar, respectively, compared to control. While non-reducing sugar was enhanced only in TAM100 from 0.74 to 0.82%, seaweed extract did not affect morphometric parameters only, but also the biochemical constituents. The sugar content of the fruit is one of the key parameters for measuring the fruit quality for marketable value. Sugars could also be used as an acceptable sensory parameter in strawberries because they are converted to organic compounds such as esters, terpenoids, and furanone [77]. Furthermore, sugar accumulation aids plants in overcoming different biotic stress, such as freezing stress, by stabilizing a variety of cellular membrane and membrane-bound organelles [78]. Seaweed extract has improved its ability to enhance the total sugar content in maize [79].

### 3.4. Bioethanol Production

**Figure 3:**
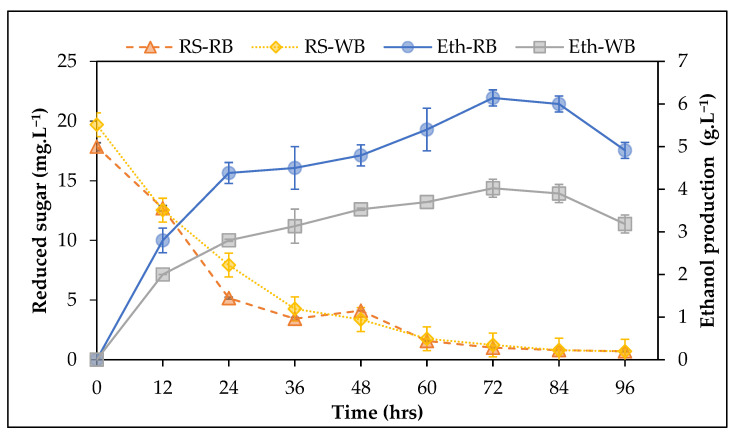
The reducing sugar consumption and bioethanol production time courses using whole seaweed biomass (WB) and residual biomass fermentation (RB). RS and Eth are reducing sugar and ethanol.

Several reports stated that acid hydrolysis is a promising saccharification technique that improves carbohydrate degradation to the monomer [15,17,80]. Hessami et al. [81] observed that H_2_SO_4_ showed the best hydrolyzation over HCI, HClO_4_, and CH_3_COOH. Kim et al. [82] investigated H_2_SO_4_ pretreatment of four marine seaweeds, *Ulva lactuca, Gelidium amansii, Laminaria japonica,* and *Sargassum fulvellum;* under high temperature and pressure, more soluble reducing sugars were produced with increasing H_2_SO_4_ concentrations. Moreover, several studies investigated that 5% H_2_SO_4_ showed the best hydrolysis effect compared to other concentrations [83]. As a result, acid hydrolysis with 5% H_2_SO_4_ was used in this study as a more efficient, fast, and cost-effective technique than enzymatic saccharification [84]. Whole seaweed biomass showed the highest reducing sugars content (19.68 g L^−1^) after hydrolysis, while the residual biomass showed 17.84 g L^−1^. The lower sugar content or residual biomass might be attributed to the loss of some sugars during the biostimulant extraction process. The obtained reducing sugars from whole and residual biomass were used as a fermentation feedstock for *S. cerevisiae*. Reducing sugars and ethanol production were monitored at 12 h intervals up to 96 h (Figure 3). As the fermentation time was increased, a gradual decrease in reducing sugars was associated with increases in bioethanol production, reaching the steady-state phase after 72 h. The maximum bioethanol production of 4.02 and 6.14 g L^−1^ was obtained at 72 h of whole biomass and residual biomass fermentation, respectively. For effective bioethanol production, cost-effective pretreatment and rapid saccharification/fermentation processes are required. The present study improved bioethanol production without additional residual biomass pretreatment. Consequently, performing TAM^®^ extraction before fermentation plays the dual role of biostimulant recovery and pretreatment for enhanced sugars recovery.

The maximum bioethanol yields regarding reducing sugars content were 0.20 and 0.34 g g^−1^ dw for the whole seaweed and residual biomass, respectively, after 72 h of fermentation. The bioethanol yield obtained from the current study is comparable to that recorded for free galactose sugar as a standard fermentation medium (0.37 g g^−1^) [48]. Kim et al. [82] demonstrated the ethanol yields of mixed sugars (glucose, galactose, xylose, L-arabinose, and mannitol) that were in the same range of our results (0.35–0.37 g g^−1^). Surprisingly, the current study’s bioethanol yield is significantly higher than many earlier reports. Osman et al. [66] obtained a bioethanol yield of 0.08 g g^−1^ from the green seaweed residue after lipid extraction using *S. cerevisiae*, and Elshobary et al. [16] obtained a bioethanol yield of 0.32 g g^−1^ from the fermentation of *Dilophus fasciola* after lipid extraction for biodiesel production. The current result confirms the efficiency of the biorefinery approach to obtain TAM^®^ biostimulant for improving plant growth and fruiting quality and utilization of the residual biomass as bioethanol feedstock, which showed the highest bioethanol production compared to the whole biomass.

## 4. Conclusions

While it has a lot of potentials to be an innovative, ecologically friendly alternative to commonly used NPK fertilizers, this study found that foliar spraying of True Algae Max, (TAM^®^) significantly improved the morpho-agronomic and bioactive qualities of strawberry when compared to routine control treatments with chemical fertilizers. Among the three practiced TAM^®^ concentrations, TAM50_%_ treatment resulted in the most efficient treatment on practically all growth metrics, including fruit yield, bioactive compounds, anthocyanin, and sugar content, with an increase reaching 110% compared to NPK control. This study shows that TAM^®^ may be a sensible tool for enhancing the morpho-agronomic and bioactive traits of strawberry plants. Furthermore, the TAM residual was suitable for bioethanol production with an ethanol yield of 0.34 g g^−1^ dw. These findings are in line with global government policies aimed at saving money, time, and effort to achieve improved lives, health, and welfare while also providing adequate economic and environmental advantages.

## 5. Patents

Seaweed extract (TrueAlgaeMax, TAM^®^) is a patent submitted at the Egyptian Patents office, Academy of Scientific Research and Technology (submission No.: 2046/2019).

## Figures and Tables

**Table 1:** Physico-chemical properties of location-soil samples during the experiment season.

Soil Parameters	Values
Particle size distribution	
Sand (%)	90.5 ± 1.6
Silt (%)	3.9 ± 0.1
Clay (%)	5.5 ± 0.6
Soil texture	Sandy
Chemical Characteristics	
pH	8.05 ± 0.03
Electrical conductivity (EC) (ds/M^−1^)	0.79 ± 0.15
Organic matter (OM) (%)	0.54 ± 0.25
Soluble Cations (mmol g^−1^ soil)	
Ca^2+^	9.00 ± 0.1
Mg^2+^	2.40 ± 0.4
Na^+^	2.15 ± 0.15
K^+^	1.10 ± 0.17
Soluble Anions (mmol L^−1^)	
HCO_3−_	1.66 ± 0.1
Cl^−^	2.20 ± 0.30
SO_4_^2−^	1.50 ± 0.25

**Table 2:** Effect of different TAM^®^ concentrations on growth characterization of strawberry.

	Treatments *	TAM0% (Control)	TAM50%	TAM100%
Parameters				
Shoot length (cm)		22.72 ± 2.93 ^a^	21.74 ± 1.43 ^b^	21.84 ± 2.30 ^b^
Increase/Decrease Rate (%)		0	−3%	−4%
Plant Fresh Weight		45.89 ± 3.58 ^b^	47.54 ± 2.65 ^a^	45.82 ± 4.80 ^b^
Increase/Decrease Rate (%)		0	10%	4%
Leaf area (Plant^−1^ cm^2^)		1856 ± 22.93 ^c^	2313.66 ± 24.56 ^a^	2252.33 ± 30.73 ^b^
Increase/Decrease Rate (%)		0.00	25%	21%
Root length (cm)		16.63 ± 1.91 ^c^	34.91 ± 2.03 ^a^	30.04 ± 1.83 ^b^
Increase/Decrease Rate (%)		0	110%	81%
Chlorophyll (mg 100 g^−1^ FW)		30.73 ± 1.25 ^c^	31.38 ± 3.44 ^b^	34.37 ± 4.03 ^a^
Increase/Decrease Rate (%)		0	2%	12%
Leaf-N (%)		1.50 ± 0.04 ^a^	1.45 ± 0.07 ^b^	1.43 ± 0.05 ^b^
Increase/Decrease Rate (%)		0	−3%	−4%
Leaf-P (%)		0.74 ± 0.02 ^a^	0.63 ± 0.01 ^b^	0.57 ± 0.03 ^c^
Increase/Decrease Rate (%)		0	−15%	−23%
Leaf-K (%)		0.87 ± 0.12 ^c^	1.01 ± 0.25 ^b^	1.34 ± 0.53 ^a^
Increase/Decrease Rate (%)		0	17%	55%

* Represented Data (*n* = 3) were mean ± SD. Different superscript letters (a > b > c) in each row indicate significant differences (*p* < 0.05).

**Table 3:** The effect of different TAM^®^ concentrations on some fruit quality of strawberry.

	Treatments *	TAM0 (Control)	TAM50	TAM100
Parameters				
Total soluble solids (TSS %)		7.58 ± 1.03 ^c^	10.12 ± 1.22 ^a^	9.60 ± 1.30 ^b^
Increase/Decrease Rate (%)		0%	33%	27%
Titratable acidity (g AC 100 g^−1^)		1.21 ± 0.28 ^a^	0.98 ± 0.05 ^b^	0.99 ± 0.10 ^b^
Increase/Decrease Rate (%)		0%	−18%	−18%
TSS/TA		6.34 ± 1.02 ^c^	10.30 ± 1.56 ^a^	9.72 ± 0.78 ^b^
Increase/Decrease Rate (%)		0%	62%	53%
Anthocyanin (mg CGE 100 g^−1^)		23.08 ± 2.91 ^b^	29.42 ± 2.53 ^a^	28.95 ± 1.99 ^a^
Increase/Decrease Rate (%)		0	27%	25%
Total phenolic (mg/g DW)		96.02 ± 2.25 ^c^	136.12 ± 3.84 ^b^	148.35 ± 3.33 ^a^
Increase/Decrease Rate (%)		0	42%	92%

* Represented Data (*n* = 3) were mean ± SD. Different superscript letters (a > b > c) in each row indicate significant differences (*p* < 0.05).

## Data Availability

Not applicable.
